# Atypical Presentation of Moyamoya Disease Presenting With Severe Headache: A Case Report

**DOI:** 10.7759/cureus.80378

**Published:** 2025-03-10

**Authors:** Faizan Khan, FNU Fatima, Faiza Malik, Nilay Bhatt, Ali Z Ansari, Shivam Gupta, Sanim A Choudhury

**Affiliations:** 1 Department of Internal Medicine, HCA Houston Healthcare Clear Lake, Webster, USA; 2 Department of Pathology and Laboratory Medicine, William Carey University College of Osteopathic Medicine, Hattiesburg, USA; 3 Department of Internal Medicine, Idaho College of Osteopathic Medicine, Boise, USA

**Keywords:** antiplatelet therapy, cerebrovascular disease, collateral vessels, computed tomography angiography (cta), coronary artery disease, headache, hypertension, moyamoya disease, stroke, vascular occlusion

## Abstract

Moyamoya disease (MMD) is a rare and progressive cerebrovascular disorder characterized by the stenosis or occlusion of the terminal portions of the internal carotid arteries, accompanied by the formation of a compensatory network of abnormal collateral vessels at the base of the brain. The disease commonly presents with transient ischemic attacks (TIAs), ischemic and hemorrhagic strokes, seizures, cognitive impairments, and headaches. While most cases manifest in childhood or early adulthood, atypical or delayed presentations have been reported. We present the case of a 56-year-old Caucasian male with a history of coronary artery disease, diabetes mellitus, and hypertension who presented with sudden-onset severe right-sided headache, left-sided numbness, and chest pain. Imaging revealed a right middle cerebral artery occlusion, consistent with MMD. The patient was managed with antiplatelet therapy and close monitoring, leading to significant improvement in symptoms. This case highlights the importance of considering MMD in patients presenting with persistent headaches and cerebrovascular symptoms and emphasizes the role of antiplatelet therapy in managing cases with preserved cerebral blood flow.

## Introduction

Moyamoya disease (MMD) is a chronic, progressive cerebrovascular disorder characterized by the gradual stenosis or occlusion of the terminal portion of the internal carotid arteries (ICAs), along with the proximal segments of the anterior and middle cerebral arteries [1]. In response to this vascular narrowing, the brain develops an extensive network of fragile collateral vessels at the base of the brain to compensate for reduced cerebral blood flow. These abnormal vessels, which appear as a "puff of smoke" on angiographic imaging, are prone to rupture, making MMD a significant cause of both ischemic and hemorrhagic strokes [2]. MMD is often distinguished from Moyamoya syndrome, which occurs secondary to other underlying conditions, such as Down syndrome, sickle cell disease, and neurofibromatosis type 1 [3]. While MMD is typically idiopathic, genetic studies have identified mutations in the RNF213 gene as a major susceptibility factor, particularly among East Asian populations where the disease is more prevalent [2]. The pathophysiological mechanisms of MMD are not fully understood, though genetic, inflammatory, and environmental factors may contribute to its development. Although MMD is most commonly diagnosed in children and young adults, it can also present in older individuals, where its symptoms may be mistaken for more common cerebrovascular diseases such as atherosclerosis. In these cases, the imaging findings may overlap, complicating the diagnosis and leading to delayed recognition. This highlights the importance of considering MMD in adults with nonspecific cerebrovascular symptoms, particularly when imaging shows collateral vessel formation or unexplained ischemic changes.

The epidemiology of MMD varies by geographic region, with the highest incidence reported in East Asian countries, such as Japan, China, and Korea [4]. However, the disease is increasingly recognized in non-Asian populations, including North America and Europe, albeit at a lower frequency. While MMD can present at any age, it follows a bimodal age distribution, with peaks of onset occurring in childhood (typically between 5 and 10 years) and adulthood (typically between 30 and 40 years) [5]. In children, MMD often manifests as transient ischemic attacks (TIAs) or ischemic strokes due to cerebral hypoperfusion, whereas, in adults, hemorrhagic strokes are more common as a result of fragile collateral vessel rupture. Additional neurological symptoms, including cognitive impairments, seizures, and persistent headaches, have been reported across all age groups. Headaches, in particular, are believed to arise from cerebral hypoperfusion, microvascular ischemia, or the dilation of collateral vessels, and they may precede major ischemic events [6]. Despite these well-documented clinical manifestations, the diagnosis of MMD is often delayed, particularly in cases presenting with nonspecific symptoms such as headaches without focal neurological deficits.

## Case presentation

A 56-year-old right-handed Caucasian male, with a history of coronary artery disease, diabetes mellitus, and hypertension, presented to the emergency department (ED) with a sudden-onset severe right-sided headache, described as the worst headache of his life. The pain was associated with left-sided numbness and chest discomfort. He denied any history of similar headaches, trauma, fever, neck stiffness, visual disturbances, or speech disturbances. His headache had been persistent for several hours before presentation, with no alleviating or exacerbating factors. The gradual onset of symptoms, including the headache and left-sided numbness, was an atypical feature, as most patients with similar complaints typically experience more acute or rapidly progressing symptoms. On arrival, the patient was alert and oriented but appeared in distress due to the severity of the headache. His vital signs were notable for elevated blood pressure at 171/83 mmHg, with a heart rate of 82 beats per minute, respiratory rate of 16 breaths per minute, and oxygen saturation of 98% on room air. Neurological examination revealed decreased sensation in the left upper and lower extremities, but motor strength, reflexes, and cranial nerve function remained intact. There was no dysarthria, facial asymmetry, or coordination deficits. Cardiovascular and respiratory examinations were unremarkable, and no murmurs or signs of heart failure were noted.

Given his medical history and symptoms, an initial working diagnosis included acute stroke, hypertensive crisis, and subarachnoid hemorrhage. Other differentials considered included reversible cerebral vasoconstriction syndrome (RCVS), vasculitis, and migraine with aura. To evaluate for vasculitis or secondary MMD, inflammatory markers, including C-reactive protein (CRP) and erythrocyte sedimentation rate (ESR), were assessed and found to be within normal limits. Additionally, autoimmune panels, including antinuclear antibodies (ANA) and antineutrophil cytoplasmic antibodies (ANCA), were negative, reducing the likelihood of an underlying inflammatory or autoimmune etiology.

**Figure 1:**
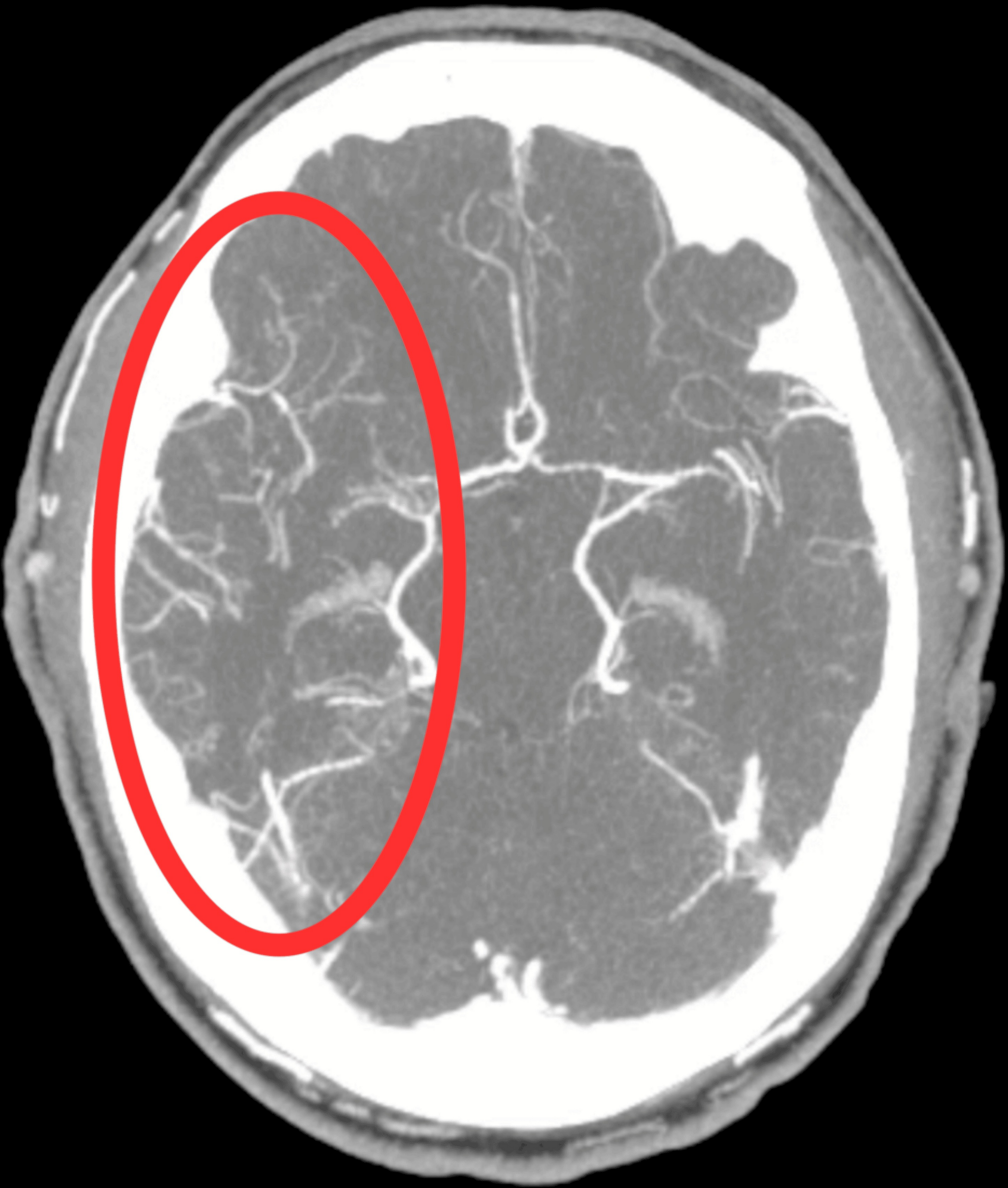
Axial view of CTA demonstrating occlusion of the right M1 segment of the middle cerebral artery (red oval), along with extensive collateral vessel formation at the base of the brain. CTA: Computed tomography angiography

**Figure 2:**
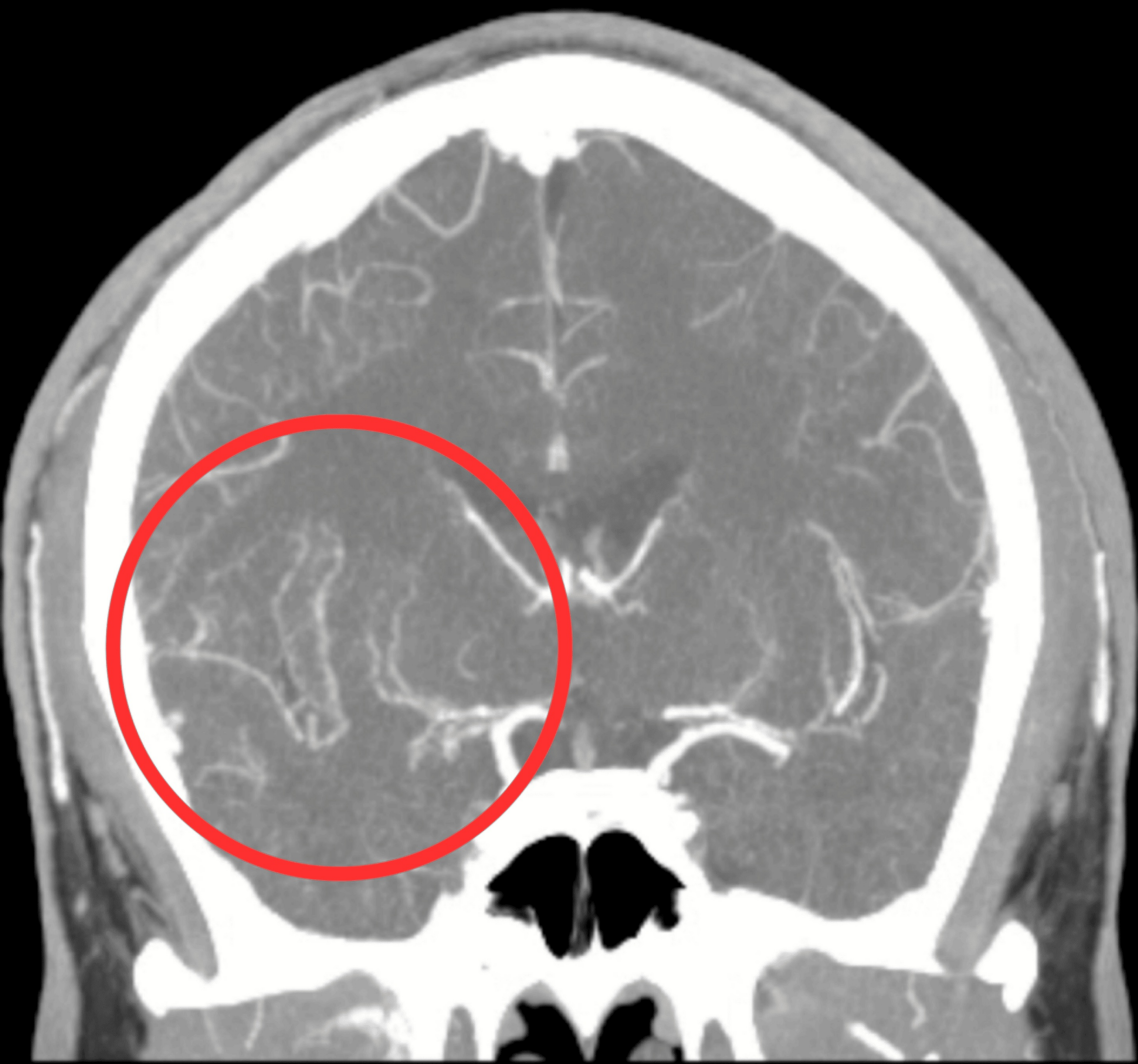
Coronal view of CTA demonstrating the occlusion of the right M1 segment of the middle cerebral artery (red circle), along with extensive collateral vessel formation. CTA: Computed tomography angiography

A non-contrast computed tomography (CT) of the head was performed, revealing low-density changes in the right posterior temporal-occipital region, consistent with an evolving infarct, but no evidence of hemorrhage. A lumbar puncture was deferred as no meningeal signs were present. A CT angiography (CTA) of the head and neck revealed mixed plaques in the carotid bifurcations bilaterally with approximately 50% stenosis at the internal carotid artery origins (Figures 1-2). More importantly, a right middle cerebral artery (RMCA) occlusion was noted, along with extensive collateral vessel formation at the base of the brain, highly suggestive of MMD. While CTA provided valuable information regarding the presence of collateral vessels, digital subtraction angiography (DSA) was not considered for further diagnostic imaging in this case. Given the clear collateral vessel formation visualized on CTA, which is typically sufficient for diagnosis, no significant involvement of the anterior cerebral arteries (ACA) or other vascular territories was noted on the CTA, and the collateral vessels were primarily confined to the middle cerebral artery territory. While hypertension and diabetes can contribute to in situ arterial stenosis with collateral formation, several factors supported the diagnosis of MMD in this case. The pattern of stenosis primarily affecting the terminal internal carotid artery with the presence of abnormal, fine collateral networks at the base of the brain (puff-of-smoke appearance) was characteristic of MMD. Furthermore, the absence of significant atherosclerotic changes in major intracranial vessels and the lack of systemic vasculitis or embolic sources strengthened the likelihood of MMD rather than atherosclerotic cerebrovascular disease.

The patient was initially managed with 325 mg of aspirin, 10 mg of labetalol for blood pressure control, and 15 mg of ketorolac tromethamine for pain management. Given the presence of ischemic findings without significant neurological deficits, neurointerventional radiology was consulted, and no urgent intervention was recommended. Instead, the patient was admitted to the intensive care unit (ICU) for close monitoring, frequent neurological assessments, and blood pressure optimization.

Over the course of his ICU stay, the patient’s symptoms gradually improved, with resolution of the left-sided numbness and a significant reduction in headache severity. A follow-up magnetic resonance imaging (MRI)/magnetic resonance angiography (MRA) confirmed the findings of chronic vascular changes consistent with MMD. Additionally, the MRI revealed evidence of chronic infarction in the RMCA territory, consistent with the earlier findings on CTA, indicating prior ischemic events in the same vascular territory. The patient has transitioned to aspirin 81 mg daily and atorvastatin 40 mg for secondary stroke prevention. Lifestyle modifications, including blood pressure control and glycemic management, were emphasized. After eight days of hospitalization, the patient was discharged in stable condition with no focal neurological deficits. He was referred to neurology for continued outpatient follow-up, with plans for serial imaging to monitor disease progression. Follow-up imaging would include a repeat CTA to assess for any changes in the vascular territories and evaluate the status of the collateral vessels. If necessary, a DSA may be considered for further evaluation of the cerebral vasculature, particularly if there are any concerns about worsening stenosis or new areas of ischemia. Additionally, repeat MRI/MRA will be performed to assess for any new ischemic changes or infarcts in the previously affected areas.

## Discussion

The delayed diagnosis of MMD in this case highlights the importance of considering this rare condition in the differential diagnosis, particularly when patients present with nonspecific symptoms such as headaches and sensory deficits. The patient's presentation of severe right-sided headache and mild left-sided numbness was atypical for MMD, as the disease typically presents with more pronounced neurological deficits, such as strokes or TIAs, especially in younger patients. In older adults, more common cerebrovascular conditions, such as ischemic stroke, hypertensive crisis, or subarachnoid hemorrhage, are often prioritized in the differential diagnosis [7]. However, other conditions must also be considered. RCVS, for example, typically presents with thunderclap headaches and focal neurological signs. Although RCVS can show constriction of multiple cerebral arteries on imaging, the absence of vasoconstriction patterns on the patient's CTA helped rule out this diagnosis. Cardioembolic strokes were also considered, particularly in patients with underlying risk factors. Cardioembolic events usually present with ischemia in different vascular territories, but the patient's clinical history and imaging findings did not support this diagnosis, as no embolic sources were identified. Intracranial artery stenosis, another potential cause, can also present similarly, especially in patients with hypertension and diabetes, who are at risk for atherosclerosis. However, the CTA revealed collateral vessel formation rather than severe stenosis or plaque buildup typically seen in large vessel atherosclerosis. Ultimately, the identification of collateral vessels on imaging was a critical diagnostic clue that led to the diagnosis of MMD, emphasizing the essential role of advanced imaging techniques such as MRI/MRA and CTA in identifying this rare condition.

MMD is a progressive disease that can lead to severe neurological deficits if left untreated. While the imaging findings of stenosis with collateral formation in this patient, along with the clinical presentation of severe headache and left-sided numbness, were highly suggestive of MMD, it is important to consider additional points in support of the diagnosis. The patient’s medical history of diabetes and hypertension, which are common risk factors for cerebrovascular disease, could raise suspicion for other conditions such as atherosclerotic disease or ischemic stroke. However, the presence of collateral vessel formation primarily in the middle cerebral artery (MCA) territory, which is characteristic of MMD, along with the lack of significant findings in other vascular territories, helped differentiate MMD from other possible causes of ischemia. Additionally, the lack of significant neurological deficits early on, despite the ischemic findings, further supported the diagnosis of MMD rather than a typical atherosclerotic stroke. The imaging findings of collateral vessel formation and the patient’s clinical course, which improved with medical management and antiplatelet therapy, were consistent with MMD. While the presence of stenosis and collateral formation in a diabetic and hypertensive individual could overlap with other cerebrovascular conditions, these factors, combined with the patient's clinical presentation, imaging findings, and response to therapy, make the diagnosis of MMD plausible. Timely diagnosis and appropriate management are critical to prevent further progression and potential complications.

In cases with preserved cerebral blood flow, as seen in this patient, medical management with antiplatelet therapy can help reduce the risk of further ischemic events [8]. While surgical revascularization remains the treatment of choice in patients with significantly impaired blood flow, this case highlights that conservative management can be effective in stabilizing patients with milder disease or less advanced hemodynamic compromise [9]. Ongoing monitoring and serial imaging are essential to assess the progression of the disease and determine if surgical intervention becomes necessary. Surgical revascularization is typically considered in patients with progressive ischemic events, worsening neurological deficits, or significant hemodynamic compromise despite medical management. Imaging findings, such as worsening stenosis or occlusion of the major cerebral arteries, development of new collateral vessels, or signs of ischemic progression, would also trigger consideration for surgical intervention. Additionally, if the patient experiences recurrent TIAs or strokes despite optimal medical therapy, surgical intervention may be indicated to restore cerebral blood flow and prevent further complications.

The pathophysiology of MMD involves progressive stenosis of the internal carotid arteries and the development of fragile collateral vessels that may predispose patients to both ischemic and hemorrhagic events. Although genetic factors are implicated in the development of MMD, environmental and other underlying conditions, such as hypertension or diabetes, may also contribute to disease progression [10]. While RNF213 gene mutations have been identified as a significant susceptibility factor, particularly in East Asian populations, genetic testing was not performed in this case. Given the patient’s presentation and the established diagnosis based on imaging, genetic confirmation was not deemed necessary for clinical management. However, genetic testing could be considered in cases with a strong family history or uncertain diagnosis to further guide risk assessment and counseling. This case serves as a reminder that MMD should not only be considered in younger patients but also in older adults, especially when presenting with unexplained cerebrovascular symptoms. Further research into the genetic and environmental factors that contribute to MMD is essential for improving early detection and tailoring treatment strategies.

## Conclusions

In this case, MMD is the most likely diagnosis, given the patient's age, medical history, and imaging findings, particularly the presence of collateral vessel formation in the right middle cerebral artery territory. While the imaging strongly supports this diagnosis, further confirmation through DSA would have provided a more definitive assessment. Differential diagnoses, such as RCVS, cardioembolic sources, and intracranial artery stenosis, should be considered in the evaluation of patients with similar symptoms. Moreover, given the patient’s condition, surgical considerations, including potential revascularization, should be closely monitored as part of ongoing management, especially if symptoms progress.
